# *Funneliformis mosseae* inoculation under water deficit stress improves the yield and phytochemical characteristics of thyme in intercropping with soybean

**DOI:** 10.1038/s41598-021-94681-9

**Published:** 2021-07-27

**Authors:** Mostafa Amani Machiani, Abdollah Javanmard, Mohammad Reza Morshedloo, Ahmad Aghaee, Filippo Maggi

**Affiliations:** 1 Department of Plant Production and Genetics, Faculty of Agriculture, University of Maragheh, P.O.Box 55136-553, Maragheh, Iran; 2 Department of Horticultural Science, Faculty of Agriculture, University of Maragheh, P.O.Box 55136-553, Maragheh, Iran; 3 Department of Plant Biology, Faculty of Agriculture, University of Maragheh, P.O.Box 55136-553, Maragheh, Iran; 4 School of Pharmacy, University of Camerino, Camerino, Italy

**Keywords:** Secondary metabolism, Drought

## Abstract

Intercropping of medicinal plants/legumes along with bio-fertilizer application is a relatively new sustainable practice for improving the yield and secondary metabolites production. Here, a 2-years field experiment was performed to evaluate the effects of water deficit stress and arbuscular mycorrhizal fungi (AMF) application (as bio-fertilizer) on nutrients concentration, dry matter yield, essential oil quantity and quality of thyme in intercropping with soybean. Three irrigation levels, including (i) irrigation after depletion of 20% (I_20_) as non-stressed, 50% (I_50_) as moderate water deficit and 80% (I_80_) available water as severe water deficit were applied as the main factor. The sub-factor was represented by different cropping patterns including thyme sole culture, replacement intercrop ratio of 50:50 and 66:34 (soybean: thyme) and the third factor was non-usage (control) and usage of AMF. According to our results, the thyme dry yield under moderate and severe water deficit stress decreased by 35 and 44% in the first year, and by 27 and 40% in the second year compared with non-stressed (I_20_) plants, respectively. Also, the macro- and micro-nutrients of thyme leaves increased significantly in intercropping patterns after application of AMF. The maximum essential oil percentage of thyme was achieved in 50:50 intercropping ratio treated with AMF. Under moderate and severe water deficits, the major constituents of thyme essential oil including thymol, *p*-cymene and *γ*-terpinene were increased in intercropping patterns treated with AMF. Generally, AMF application in intercropping ratio of 50:50 may be proposed to farmers as an eco-friendly approach to achieve desirable essential oil quality and quantity in thyme under water deficit stress conditions.

## Introduction

Medicinal and aromatic plants are the reservoir of secondary metabolites (including essential oil and polyphenolic compounds) which are useful remedies for humans and also have a great importance in fine chemicals, pharmaceuticals, cosmetics, perfumery and food industry. More than 80% of population in developing countries, especially Iran, use medicinal and aromatic plants in their healthcare system^1^. Thyme (*Thymus vulgaris* L.), belonging to Lamiaceae family, is a flowering perennial plant native to the Mediterranean regions, including North Africa, Southern Europe and Western Asia, and adaptable to a wide range of environmental conditions^2^. Thyme has a great economic importance worldwide, especially for the production of its essential oil which has been estimated in 50–100 ton/year. The essential oil extracted form thyme has been reported to be as one of the top 10 essential oils for sale worldwide^3^. The aerial parts as well as the volatile constituents of thyme are used in the folk medicine to treat cough, diabetes, cold and chest infections due to the documented antiseptic, antibacterial, antifungal, antispasmodic, antitussive, expectorant and analgesic properties related mainly to the presence of the phenolic monoterpenes thymol and carvacrol^4,5^. Besides these compounds, the thyme essential oil is also characterized by the presence of *ɣ*-terpinene and *p*-cymene which are recognized as the biogenetic precursors of thymol and carvacrol^6^.

The climate-changes around the world indicate clearly that annual maximum temperature and the aridity are increasing^7^. Water deficit stress, known as one of the limiting abiotic factors, affects every aspect of the plant growth, morphology, physiology and productivity in the arid and semi-arid regions^8,9^. In medicinal and aromatic plants, increasing the essential oil content is considered as one of main defense mechanisms against drought stress conditions, because in case of stress, more metabolites are produced in the plants to prevent cell oxidization^10^.

In addition, the nutrient use efficiency (NUE) of chemical fertilizer is reduced under drought conditions due to the decrease of mobility and low rate of mineral diffusion. Thus, the lower NUE along with the negative effects given by the chemical fertilizer application (e.g., soil, air and water pollution, soil acidification, mineral depletion, decrease of soil micro-organisms and harmful effects on human health) forced the agrochemical companies to replace the chemical inputs with eco-friendly fertilizers. Recently, the interaction among plants and soil microbial population is a conventional method to limit the demand of synthetic fertilizers over sustainable agricultural systems. Arbuscular mycorrhizal fungi (AMF) are soil borne micro-organisms forming a mutualistic symbiotic association with more than 80% of plant species^11,12^. The coexistence between AMF and host plants improves the conditions for an optimal plant growth. In medicinal and aromatic plants, previous research indicated that inoculation of AMF in dill (*Anethum graveolens* L.), rose geranium (*Pelargonium graveolens* L.) and *Mentha* × *piperita* improves the nutrient uptakes and essential oil productivity^13,14^.

Conventional agricultural systems are based on cultivation and productivity using sole plant cultures with intensive application of chemical inputs including synthetic fertilizer, pesticides, herbicides and others^15^. The expansion of intensive crop production systems for long terms caused several problems, such as the reduction of biodiversity and the environmental contamination. The increasing concern regarding the negative impacts of these conventional agricultural methods on the ecosystems suggests the exploration of alternative methods endowed with improved efficiency and sustainability as well as high productivity^16^.

The intercropping system is one of the well-known sustainable and eco-friendly agricultural methods in which two or more plants are grown simultaneously. This provides interconnections between plants leads to more efficient use of environmental resources including nutrients, water, radiation, land and also reduced damages of pests and plant diseases^17,18^. Among the various intercropping systems available, intercropping based on legume species provides an added value in order to minimizing the adverse impact of chemical fertilizer (due to nitrogen transfer from legume to companion crops or N as an input for subsequent crops)^19,20^.

Iran is basically an arid country, with an average annual rainfall ranging from 224 to 275 mm/year (70% less than global average)^21^. Recently, a considerable demand for thyme and its different products has been registered around the world. In order to comply with this objective, the improvement of thyme productivity based on the application of sustainable strategies that increase nutrient uptake under water scarcity conditions, especially in the arid and semi-arid regions of the world such as Iran, is urgently needed. Thus, an experiment was aimed to compare the impacts of different water deficit levels and application of AMF on the dry matter yield, nutrients uptake, essential oil quantity and quality of thyme in different intercropping patterns with soybean.

## Materials and methods

### Area of study

The experiments were performed over two successful growing year (April–October 2018 and April–October 2019) at the Maragheh University research farm, East Azerbaijan Province, Iran. The filed soil had a sandy clay loam texture with pH of 7.53; the electrical conductivity (EC) and the TOC (total organic-carbon) content was 1.216 dS/m and 0.76%, respectively. The contents of macro- and micro-elements and physico-chemical characteristics are listed in Table 1. Also, the monthly weather data in the research area are provided in Table 2.

**Table1:** Physico-chemical properties of field soil (depth of 0–30 cm) an average over the 2 years.

Soil texture	Sand (%)	Silt (%)	Clay (%)	Field capacity (%)	Permanent wilting point (%)	Organic matter (%)	EC (ds/m)	pH	Amount of cation exchange capacity (Cmolc/kg)	Amount potassium (mg/kg)	Available phosphorus (mg/kg)	Total nitrogen (%)
Sandy clay loam	47	24	29	27.4	13.9	0.76	1.216	7.53	25.8	516.3	12.6	0.09

**Table 2:** Monthly average temperature and total monthly precipitation of experimental site during two growing seasons and long-term averages.

Year	April	May	June	July	August	September	October
**Monthly average temperature (°C)**							
2018	12.6	16.6	24.1	30.2	27.7	23.6	15.9
2019	10.4	18.5	25.7	27.6	27.8	22.1	16.7
2-year mean	11.5	17.5	24.9	28.9	27.8	22.8	16.3
10-year mean	12.7	18.0	24.0	27.9	27.4	22.0	15.1
**Total monthly precipitation (mm)**							
2018	44.9	54.5	1.7	0.1	0	0.2	19.5
2019	51.3	37.8	4.2	0.0	0.0	0.0	6.3
2-year mean	48.1	46.2	3.0	0.1	0.0	0.1	12.9
10-year mean	39.4	16.6	3.4	0.4	0.3	1.7	17.1

### Treatments

**Figure 1:**
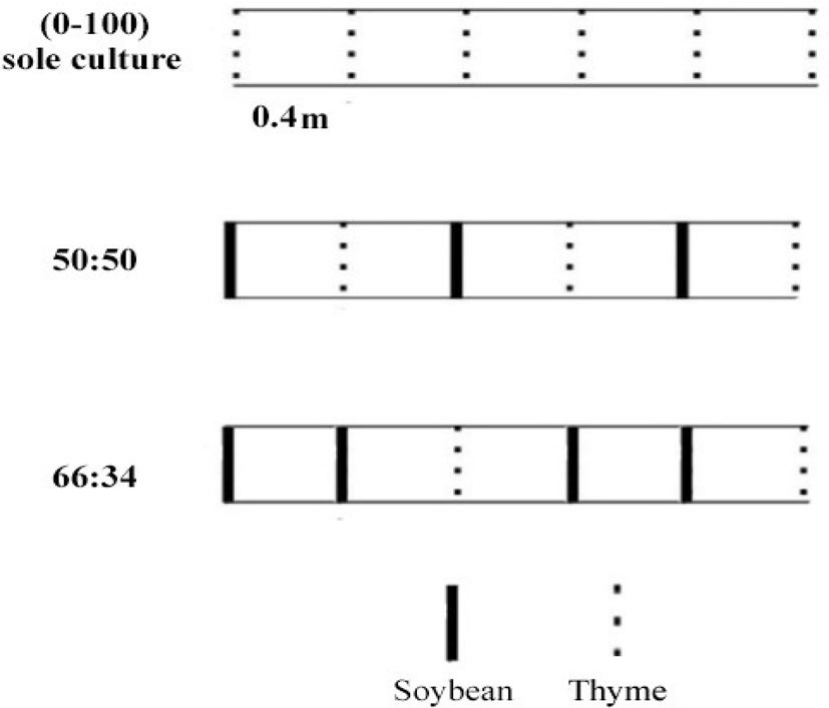
Sketch of soybean/thyme planting patterns.

A split plot experiment, based on a RCBD design with three replications and 18 treatments, was performed. The main factor was represented by different water deficit stress levels, including (i) irrigation after depletion of 20% available water (I_20_) as non-stressed, (ii) 50% of available water (I_50_) as moderate water deficit, and (iii) 80% available water (I_80_) as severe water deficit. The sub-factor was different cropping patterns, including (i) thyme sole culture (T_s_) and (ii) soybean–thyme replacement intercropping series (in proportion of 50:50 and 66:34). The experimental treatments based on proportions of soybean–thyme intercrops pattern is given in Table 3. In addition, the third factor was represented by (i) non-application (control) and (ii) application of AMF as bio-fertilizer. Thyme and soybean were sown in 4 m long rows and the distance of each row was set to 0.4 m (Fig. 1). Also, a 1.5 m distance was maintained between different treatments in order to exclude any influence of lateral water movement. For AMF treatments, the mycorrhizal fungal spores (*Funneliformis mosseae*; 1000 spore/10 g soil) were added to the soil at planting (Zist Fanavar Sabz Company, Iran). Based on the soil physico-chemical analysis, in all treatments, 60 kg ha^−1^ superphosphate triple and 60 kg ha^−1^ N (30 kg ha^−1^ as starter, 30 kg ha^−1^ in vegetative growth) were added into the soil before planting and during planting, respectively. Thyme Seedlings were obtained from Pakan Bazr company, Isfahan, Iran and sown on 15 April 2018 with density of 20 plants per row and 20 cm row distance. Before sowing, the seeds of soybean cultivar Sari (obtained from Mazandaran Agricultural and Natural Resources Research) were inoculated with *Rhizobium japonicum* and sown on 5 April 2018 and 10 April 2019 in both growing years. The plant material and seeds were obtained under the supervision and permission of Maragheh University and according to national guidelines and all authors comply with all the local and national guidelines. In order to reduce the plant mortality, in the first month of growth the plants were watered normally. Water stress treatments were performed according to the maximum allowable depletion (MAD) percentage of the soil available water (SAW). The water stress treatments applied were 20, 50 and 80% MAD of SAW, including the non-stress (I_20_), moderate water deficit (I_50_) and severe water deficit (I_80_), respectively. For measuring the soil water content, a Time-Domain Reflectometry (TDR) probe (Model TRIME-FM, England) was used. The readings were performed at the center of each experimental plot at a depth of 30 cm. The following equations was used for calculating the irrigation depth^22^:

$${\text{SAW}} = \, (\uptheta_{{{\text{fc}}}} - \, \uptheta_{{{\text{pwp}}}} ) \, \times {\text{ d }} \times { 1}00,$$

$${\text{I}}_{{\text{d}}} = {\text{ SAW }} \times {\text{ p,}}$$

$${\text{I}}_{{\text{g}}} = \, \left[ {{\text{I}}_{{\text{d}}} \times { 1}00} \right] \, /{\text{ E}}_{{\text{a}}} .$$

In these equations, SAW, θ_fc_, θ_pwp_, d, I_d_, p, E_a_ and I_g_ are: soil available water, soil field capacity (27.4%), permanent wilting point (13.9%), soil layer depth (cm), irrigation depth (cm), fraction of SAW (20%, 50% and 80%) that can be depleted from the root zone, irrigation efficiency (%) averagely assumed 65%^23^ and the gross depth of irrigation (cm), respectively.

**Table 3:** Experimental treatments based on proportions of soybean–thyme intercrops.

Soybean density (m^2^)	Thyme density (m^2^)	Proportion of thyme-soybean intercrops (%)	Proportion of thyme/soybean intercrops (plant m^2^)
45	12.5	0–100 (sole culture)	12.5
45	12.5	50–50	23–6
45	12.5	66–34	30–4

### Measurements

#### Dry yield

At full flowering stage, the thyme aerial parts were harvested randomly on 15 August 2018 and 27 August 2019. The plants were harvested from a 1.6 m^2^ area (middle line of each treatment). After harvesting, the samples were dried at room temperature for two weeks and the dry matter yield was recorded.

#### Mycorrhizal fungal colonization

To determine the root colonization percentage, the young fresh roots of thyme were collected from the depth of 10–20 cm of the soil. The root colonization was investigated according to the method described by Phillips and Hayman^24^. Firstly, the roots were washed with water and then cut into the small pieces (1.5 cm length) and followed by in 10% potassium hydroxide at 90 °C for 10 min. Afterwards, the segments were placed in 2% HCl at 25 °C for 10 min and then stained with trypan blue (0.05%) and lactic acid (80%) for 12 h. Eventually, the roots were washed and stored in lactic acid, glycerol and distilled water (lactic acid and glycerol in proportions of 1:1:1 v/v/v) solution. Finally, the colonization percentage of thyme root was measured according to the method of Giovannetti and Mosse^25^.

#### Essential oil content and chemical analyses

40 g of dried aerial parts of herbs were roughly grounded and placed in a 1 L flask where 400 mL of distilled water and conventional hydro-distillation for 3 h, using a Clevenger-type apparatus, was performed. In order to remove possible water drops, Na_2_SO_4_ was added to each extracted essential oil and kept at 4 °C in the refrigerator before analysis. For calculating the essential oil content and yield of thyme, the following equations were used:

$$\begin{aligned} & {\text{Essential}}\,{\text{oil}}\,{\text{content}}\,{\text{of}}\,{\text{thyme}} = ({\text{distilled}}\,{\text{essential}}\,{\text{oil}} \,\, ({\text{g}})/40\,{\text{g}}) \times 100\, \\ & {\text{Essential}}\,{\text{oil}}\,{\text{yield}}\,{\text{of}}\,{\text{thyme}}\,({\text{g}}\,{\text{m}}^{ - 2} ) = {\text{dry}}\,{\text{yield}}\,{\text{of}}\,{\text{thyme}}\,\,({\text{g}}\,{\text{m}}^{ - 2} ) \times {\text{essential}}\,{\text{oil}}\,{\text{content}}\,\,(\% ). \\ \end{aligned}$$

The thyme essential oil constituents were analyzed using GC–FID and GC–MS instruments. GC–MS analysis was performed in an Agilent gas chromatograph (7990B) coupled to a 5977A mass spectrometer. The separation was done in a HP-5 MS capillary column (5% Phenyl Methylpolysiloxane, 30 m length, 0.25 mm i.d., 0.25 µm film thickness). The following oven temperature was set up for the column: 10 min at 60 °C, then the temperature raised with the rate of 3 °C/min to 250 °C, finally held for 15 min at 250 °C. The carrier gas (Helium) was used at a flow rate of 1 mL/min. Electron-impact (EI) was set to be 70 eV. The injector split ratio was 1:50 and mass range acquisition was from 40 to 400 *m/z*. Gas chromatographic (GC-FID) separations was performed in an Agilent technology instrument (Agilent 7990B, USA), coupled with a flame ionization detector (FID). VF-5MS column used in the GC-FID device had the same stationary phase and dimensions as the HP-5MS one. The oven temperature program was the same used for GC–MS analysis. Injector and detector temperatures were set at 230 and 240 °C, respectively. Diluted essential oils (1: 100 in *n*-hexane) were used for the injection. The peak areas were normalized without using correction indices.

To identify essential oil constituents, a combinational procedure was performed including determination of arithmetic retention indices using coherence of homologous series of hydrocarbons (Supelco, Bellefonte, USA), comparing retention indices with those reported in the reference literature^26^, and the computer matching of mass acquisition with the WILEY275 and NIST 05 libraries.

#### Plant nutrient concentration

Thyme leaf samples were analyzed for nutrients concentrations. The Kjeldahl method was used to determine the N content. The concentration of K was also measured using flame photometry^27^. P was determined by the yellow method^28^. The content of P was determined at 470 nm using a spectrophotometer. An atomic absorption spectrophotometer (AA-6300 F; Shimadzu, Japan) was used to determine the concentration of micro-nutrients including Ca, Fe and Mg of each samples.

### Statistical analysis

The homoscedasticity and normality test of all data were checked with Levene test and Anderson–Darling methods, respectively. Analysis of variance (ANOVA) and the means comparison (compared with LSD at 5% probability level) were performed using the SAS software version 9.1.

## Results

### Root colonization percentage

**Figure 2:**
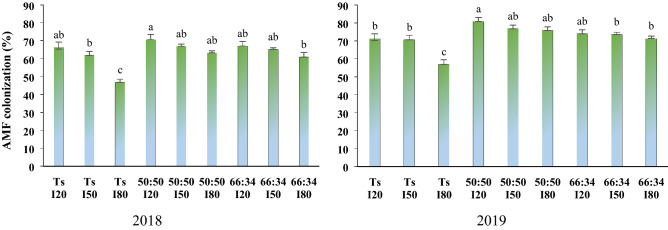
The root AMF colonization of in different water deficit levels and planting patterns.

In both years, the highest root colonization percentage was measured in the 50:50 intercrop ratio with non-stressed plants (I_20_). Also, the lowest percentage of thyme root colonization (46.81% in the first year and 56.87% in the second year) was observed in the sole culture of thyme under severe stress (I_80_). The colonization rate in moderate (I_50_) and severe water deficit (I_80_) decreased by 5 and 16% in the first year, and 2 and 10% in the second year, respectively, in comparison with I_20_ (Fig. 2).

### Dry yield

**Figure 3:**
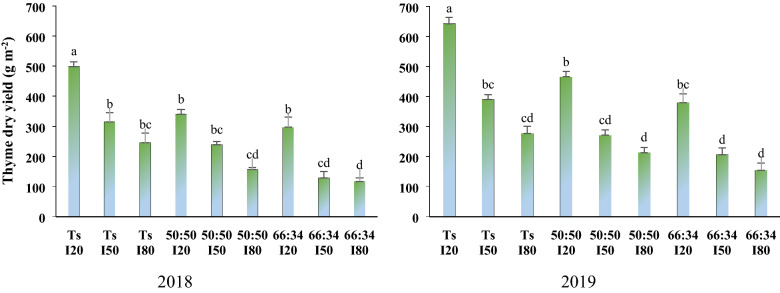
The dry yield of thyme in different water deficit levels and planting patterns.

The dry matter yield of thyme was significantly impacted by single effect of three factors and interaction of cropping patterns × water deficit levels. The highest and lowest dry yield (in both years) was achieved in sole culture of thyme with well-watered condition (I_20_) and intercrop ratio of 66:34 with I_80_ water deficit (Fig. 3). In the first and second year, application of AMF enhanced the dry productivity by 17.3 and 14.5%, respectively, in comparison with control. Furthermore, the thyme dry yield in the moderate (I_50_) and severe water deficit (I_80_) decreased by 35 and 44% in the first year, and by 27 and 40% in the second year, respectively, in comparison with I_20_ (Table 4).

**Table 4:** The dry matter yield, essential oil content and yield of thyme in different cropping patterns and irrigation levels with AMF application.

	Treatments	Dry yield (g/m^2^)		Essential oil content (%)		Essential oil yield (g/m^2^)	
		2018	2019	2018	2019	2018	2019
Cropping pattern	T_s_	419.3 a	533.7 a	1.13 c	1.78 c	4.7 a	9.4 a
	50:50	244.8 b	331.2 b	1.62 a	2.31 a	3.9 b	7.6 b
	66:34	175.1 c	219.5 c	1.32 b	1.95 b	2.2 c	4.2 c
Fertilization	Control	257.52 b	337.1 b	1.28 b	1.95 b	3.07 b	6.35 b
	AMF	301.96 a	385.8 a	1.42 a	2.08 a	4.09 a	7.81 a
Drought stress	I_20_	379.01 a	465.9 a	1.11 c	1.78 b	4.11 a	8.17 a
	I_50_	246.21 b	340.8 b	1.69 a	2.18 a	3.98 a	7.42 a
	I_80_	213.99 b	277.7 c	1.26 b	2.09 a	2.66 b	5.63 b
Significance	C	**	**	**	**	**	**
	F	**	**	**	**	**	**
	D	**	**	**	**	**	**
	C × F	ns	ns	*	*	ns	ns
	C × D	*	**	ns	ns	**	**
	D × F	ns	ns	ns	ns	ns	ns
	C × D × F	ns	ns	ns	ns	ns	ns

Ts, 50:50 and 66:34 indicating thyme sole culture and intercropping ratio of thyme with soybean; I_20_, I_50_ and I_80_ indicating non-stressed, moderate and severe water deficit levels.

ns, * and ** indicated no significant difference, significant at 5% probability level and significant at 1% probability level, respectively.

### Nutrient concentrations

Most of macro- and micronutrient concentrations was significantly impacted by water deficit levels, cropping patterns and AMF application. In both years, the maximum and minimum concentration of N, K, Ca and Fe was obtained in intercrop ratio of 50:50 and thyme sole culture. In addition, the highest content of Mg was recorded in intercrop ratio of 66:34. Base on the average of both years, the concentration of N, P, K, Ca, Mg and Fe with AMF application enhanced by 10, 31, 14, 8, 17 and 16, respectively, in comparison with control. Among water deficit levels, the highest and lowest concentration of micro and macro-nutrients was achieved in non-stressed plants and severe water deficit conditions (Table 5).

**Table 5:** The macro- and micro-nutrients concentration of thyme in different treatments.

Treatments	N content (g/kg)		P content (g/kg)		K content (g/kg)		Ca content (g/kg)		Mg content (g/kg)		Fe content (mg/kg)		Zn content (mg/kg)	
	2018	2019	2018	2019	2018	2019	2018	2019	2018	2019	2018	2019	2018	2019
**Cropping pattern**														
Sole culture	17.1 b	18.7 c	1.95 a	2.1 a	13 b	14.6 b	4.2 ab	5.1 b	2.4 b	2.9 b	310.1 b	401.5 c	18.04 a	21.2 b
50:50	19.6 a	22.5 a	2.05 a	2.3 a	15.2 a	17.1 a	4.4 a	5.3 a	2.5 ab	3.3 a	377.8 a	503.8 a	18.96 a	23.3 a
66:34	18.6 a	21.1 b	1.96 a	2.2 a	15.1 a	16.7 a	4.1 b	5.2 ab	2.6 a	3.4 a	329.7 b	448.2 b	18.27 a	20.8 b
**Fertilization**														
Control	17.5 b	19.8 b	1.6 b	1.9 b	13.5 b	15.1 b	3.98 b	5.04 b	2.3 b	2.98 b	311.1 b	420.5 b	17.87 b	20.9 b
AMF	19.4 a	21.7 a	2.1 a	2.5 a	15.4 a	17.1 a	4.43 a	5.33 a	2.8 a	3.40 a	367.3 a	481.8 a	18.99 a	22.6 a
**Drought stress**														
I_20_	22.25 a	24.7 a	2.35 a	2.82 a	18.02 a	19.9 a	4.8 a	5.8 a	3.04 a	3.66 a	408.2 a	532.8 a	20.9 a	25.1 a
I_50_	18.1 b	20.2 b	1.96 b	2.3 b	14.1b	15.7 b	4.2 b	5.1 b	2.51 b	3.16 b	318.2 b	436.7 b	18.5 b	21.5 b
I_80_	15.04 c	17.4 c	1.65 c	1.99 c	11.29 c	12.8 c	3.7 c	4.7c	2.05 c	2.75 c	291.2 b	384.1 c	15.9 c	18.7 c
**Significance**														
C	**	**	ns	ns	**	**	*	*	*	**	**	**	ns	**
F	**	**	**	**	**	**	**	**	**	**	**	**	*	**
D	**	**	**	**	**	**	**	**	**	**	**	**	**	**
C × F	ns	ns	ns	ns	ns	ns	ns	ns	ns	ns	ns	ns	ns	ns
C × D	ns	ns	ns	ns	ns	ns	ns	ns	ns	ns	ns	ns	*	*
D × F	ns	ns	ns	ns	ns	ns	ns	ns	ns	ns	ns	ns	ns	ns
C × D × F	ns	ns	ns	ns	ns	ns	ns	ns	ns	ns	ns	ns	ns	ns

Ts, 50:50 and 66:34 indicating thyme sole culture and intercropping ratio of thyme with soybean; I_20_, I_50_ and I_80_ indicating non-stressed, moderate and severe water deficit levels.

ns, * and ** indicated no significant difference, significant at 5% probability level and significant at 1% probability level, respectively.

### Essential oil content

**Figure 4:**
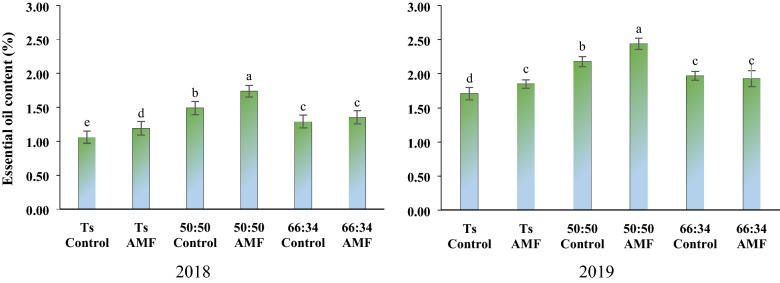
The essential oil percentage of thyme in different planting patterns and application of AMF.

AMF application, different cropping patterns and water deficit levels and interaction of cropping patterns × AMF had a significant impact on the essential oil content of thyme. Among three levels of water deficit, the maximum essential oil productivity was measured in I_50_ water scarcity that was 52% (in 2018) and 22% (in 2019) higher than those observed in I_20_, respectively (Table 4). Based on the interaction of cropping patterns × AMF application, the highest essential oil content was achieved in intercropping ratio of 50:50 after AMF application. Also, the lowest essential oil content was observed in the sole culture of thyme without usage of AMF (Fig. 4).

### Essential oil yield

**Figure 5:**
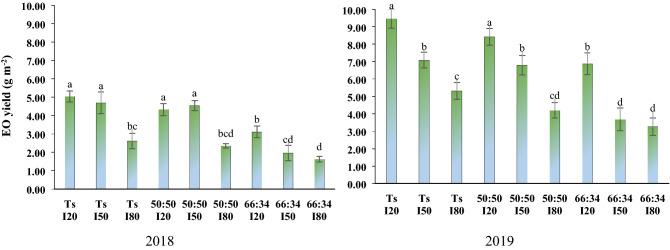
The essential oil yield of thyme in different water deficit levels and planting patterns.

AMF application, different cropping patterns and water deficit levels and interaction of cropping patterns × water deficit had a significant impact on the essential oil yield of thyme. In the first and second year, application of AMF increased the essential oil yield by 59.6 and 23%, respectively, in comparison with control (non-AMF). Based on the interaction of cropping patterns × water deficit, the highest essential oil yield was achieved in the non-stressed plants (I_20_) in sole culture (Fig. 5). Finally, the essential oil yield in I_50_ and I_80_ water deficit stresses decreased by 3 and 35% in the first year, and by 9 and 31% in the second year, respectively, when compared with well-watered plants (I_20_) (Table 4).

### Essential oil composition

In total, 29 compounds were identified in the thyme essential oils, accounting for 97.45–99.45% and 97.10–99.77% of the total compositions at the first and second year, respectively (Tables 6 and 7).

**Table 6:** Proportion of thyme essential oil constituents in different cropping patterns and irrigation levels with AMF application (first year).

No	Components	RI^a^	LIT RI^b^	Treatments^c^																		ID^d^
				T_s_ M_0_ I_20_	T_s_ M_0_ I_50_	T_s_ M_0_ I_80_	T_s_ M_1_ I_20_	T_s_ M_1_ I_50_	T_s_ M_1_ I_80_	50:50 M_0_ I_20_	50:50 M_0_ I_50_	50:50 M_0_ I_80_	50:50 M_1_ I_20_	50:50 M_1_ I_50_	50:50 M_1_ I_80_	66:34 M_0_ I_20_	66:34 M_0_ I_50_	66:34 M_0_ I_80_	66:34 M_1_ I_20_	66:34 M_1_ I_50_	66:34 M_1_ I_80_	
1	*α*-Thujene	921	924	0.54	0.69	0.59	0.62	0.54	0.67	0.62	0.52	0.69	0.53	0.36	0.77	0.51	0.47	0.74	0.42	0.55	0.64	RI-MS
2	*α*-Pinene	931	932	0.88	0.73	0.79	0.75	0.53	0.66	0.64	0.32	0.68	0.57	0.27	0.51	0.67	0.47	0.55	0.20	0.64	0.49	Std
3	Camphene	944	946	6.89	7.21	7.12	7.42	8.29	7.92	6.52	7.11	7.54	8.51	7.73	7.62	7.23	7.90	7.96	8.41	7.23	6.88	Std
4	Sabinene	968	969	0.67	0.56	0.54	0.18	0.12	0.16	0.74	0.65	0.57	0.24	0.16	0.07	0.78	0.52	0.62	0.19	0.18	0.14	Std
5	1-Octen-3-ol	972	974	0.24	0.22	0.14	0.26	0.18	0.15	0.09	0.05	0.19	0.13	0.09	0.23	0.22	0.21	0.19	0.33	0.24	0.24	RI-MS
6	*β*-Myrcene	985	988	2.26	1.79	2.12	2.31	2.04	2.11	2.15	1.74	1.83	2.04	2.02	1.98	1.97	2.16	2.22	1.72	1.94	1.88	Std
7	3-Octanol	997	998	tr	–	0.04	–	–	tr	–	–	–	–	tr	–	tr	–	–	–	–	–	RI-MS
8	*α*-Terpinene	1011	1014	0.69	0.49	0.43	0.67	0.43	0.69	0.72	0.34	0.52	0.75	0.09	0.39	0.66	0.37	0.41	0.86	0.48	0.62	RI-MS
9	***p*-Cymene**	**1019**	**1020**	**11.74**	**12.37**	**12.96**	**13.04**	**12.90**	**13.61**	**12.78**	**12.04**	**13.81**	**13.17**	**12.15**	**13.87**	**12.94**	**12.06**	**13.57**	**12.83**	**12.37**	**13.92**	Std
10	Limonene	1022	1024	0.51	0.45	0.26	0.49	0.43	0.19	0.39	0.19	0.53	0.50	0.23	0.22	0.37	0.27	0.35	0.58	0.40	0.43	RI-MS
11	1,8-Cineole	1024	1026	5.83	4.74	5.05	4.62	3.67	3.84	5.12	4.61	4.78	4.27	2.94	3.57	5.22	4.63	4.59	4.47	3.19	4.14	Std
12	***γ*-Terpinene**	**1052**	**1054**	**13.40**	**15.12**	**14.49**	**13.54**	**16.07**	**13.83**	**14.64**	**17.22**	**17.10**	**15.59**	**17.03**	**17.48**	**13.89**	**16.88**	**15.73**	**14.12**	**14.71**	**14.96**	Std
13	*cis*-Sabinene hydrate	1064	1065	0.53	0.59	0.61	0.41	0.63	0.54	0.47	0.36	0.39	0.40	0.28	0.19	0.37	0.43	0.48	0.26	0.36	0.21	RI-MS
14	*α*-Terpinolene	1066	1068	0.08	0.14	0.18	0.15	0.16	0.21	0.11	0.11	0.16	0.11	0.09	0.18	0.14	0.12	0.09	0.07	0.14	0.22	RI-MS
15	Linalool	1094	1095	3.17	2.43	2.16	2.19	1.71	1.83	2.91	1.88	2.08	1.87	1.71	1.37	2.37	2.04	1.98	1.60	1.27	1.58	Std
16	Camphor	1137	1141	5.37	5.14	4.62	5.61	5.16	5.60	4.96	4.32	3.74	4.21	4.61	4.12	5.22	4.55	5.12	4.88	4.26	4.89	RI-MS
17	Borneol	1161	1165	1.19	1.07	0.84	1.35	1.11	1.21	0.96	1.11	0.93	1.20	1.09	0.92	1.05	1.08	0.76	1.33	0.96	1.17	Std
18	Terpinen-4-ol	1171	1174	0.19	0.21	0.34	0.44	0.58	0.51	0.22	0.56	0.44	0.36	0.50	0.74	0.42	0.32	0.49	0.57	0.81	0.46	RI-MS
19	Carvacrol, methyl ether	1238	1241	1.27	1.37	1.23	1.12	1.31	1.16	1.19	1.52	1.43	1.13	1.39	1.19	1.40	1.44	1.38	1.21	1.48	1.39	RI-MS
20	Geranial	1260	1264	0.16	0.09	0.14	0.20	0.08	0.14	0.07	tr	0.04	0.13	0.09	0.08	0.06	0.11	0.09	0.16	0.11	0.09	RI-MS
21	**Thymol**	**1287**	**1289**	**31.12**	**32.51**	**33.10**	**31.19**	**32.12**	**34.19**	**33.52**	**36.91**	**31.89**	**32.12**	**37.26**	**34.79**	**33.10**	**34.51**	**31.94**	**33.85**	**36.62**	**35.74**	Std
22	Carvacrol	1297	1298	2.13	2.78	2.35	2.45	2.63	2.01	2.39	2.73	2.94	2.71	2.83	3.18	2.26	2.83	2.55	2.51	3.41	2.83	Std
23	Thymol acetate	1347	1349	0.49	0.44	0.51	0.29	0.18	0.26	0.53	0.58	0.47	0.21	0.24	0.25	0.46	0.54	0.49	0.24	0.38	0.27	RI-MS
24	(*E*)-Caryophyllene	1415	1417	3.81	3.66	3.54	4.19	3.66	3.98	3.91	2.72	3.65	3.91	2.72	2.71	4.08	2.57	3.15	4.12	3.43	3.19	Std
25	*α*-Humulene	1451	1452	tr	–	–	–	–	tr	0.06	–	–	–	–	–	–	0.04	–	–	–	tr	RI-MS
26	Germacrene D	1481	1484	0.39	0.32	0.26	0.38	0.21	0.11	0.44	0.19	0.25	0.56	0.17	0.26	0.49	0.12	0.21	0.37	0.16	0.12	RI-MS
27	δ-Cadinene	1521	1522	0.79	0.63	0.66	0.61	0.44	0.37	0.54	0.37	0.42	0.58	0.21	0.29	0.56	0.51	0.44	0.49	0.44	0.33	RI-MS
28	Caryophyllene oxide	1581	1582	2.04	1.72	1.97	1.86	1.73	1.91	1.76	1.53	1.61	1.67	1.24	1.28	2.12	1.62	1.83	1.63	1.69	1.71	Std
29	β-Bisabolene	1766	1768	–	–	tr	–	–	–	–	–	–	–	–	–	–	–	–	–	–	–	RI-MS
Total identified (%)				97.45	98.47	98.04	98.84	98.91	98.93	99.45	98.5	98.74	97.87	99.33	99.12	98.56	98.77	97.93	99.42	98.95	99.12	
**Grouped compounds (%)**																						
Monoterpene hydrocarbons				37.58	39.41	39.3	41.02	42.35	38.84	39.2	39.13	42.33	41.3	40.44	43.77	38.02	41.1	42.15	40.33	40	40.52	
Oxygenated monoterpenes				52.53	52.51	52.13	50.52	50.34	53.51	53.45	54.21	50.29	49.72	54.43	50.58	53.07	52.6	49.96	52.15	52.99	52.98	
Sesquiterpene hydrocarbons				5.03	4.61	4.46	5.18	4.31	4.49	4.95	3.28	4.32	5.05	3.10	3.26	5.13	3.24	3.80	4.98	4.03	3.67	
Oxygenated sesquiterpenes				2.04	1.72	1.97	1.86	1.73	1.91	1.76	1.53	1.61	1.67	1.24	1.28	2.12	1.62	1.83	1.63	1.69	1.71	
Others				0.27	0.22	0.18	0.26	0.18	0.18	0.09	0.05	0.19	0.13	0.12	0.23	0.22	0.21	0.19	0.33	0.24	0.24	

^a^RI, linear retention indices on DB-5 MS column, experimentally determined using homologue series of *n*-alkanes.

^b^Relative retention indices taken from Adams.

^c^Ts, 50:50 and 66:34 indicating thyme sole culture and intercropping ratio of thyme with soybean; I_20_, I_50_ and I_80_ indicating non-stressed, moderate and severe water deficit levels; M_0_: non-usage of AMF, M_1_: usage of AMF.

^d^Identification methods: MS, by comparison of the mass spectrum with those of the computer mass libraries Wiley, Adams and NIST 08; RI, by comparison of retention index with those reported in Adams and NIST 08; Std, by comparison of the retention time and mass spectrum of available authentic standard. Bold values show the major constituents of thyme essential oil.

**Table 7:** Proportion of thyme essential oil constituents in different cropping patterns and irrigation levels with AMF application (second year).

No	Components	RI^a^	LIT RI^b^	Treatments^c^																		ID^d^
				T_s_ M_0_ I_20_	T_s_ M_0_ I_50_	T_s_ M_0_ I_80_	T_s_ M_1_ I_20_	T_s_ M_1_ I_50_	T_s_ M_1_ I_80_	50:50 M_0_ I_20_	50:50 M_0_ I_50_	50:50 M_0_ I_80_	50:50 M_1_ I_20_	50:50 M_1_ I_50_	50:50 M_1_ I_80_	66:34 M_0_ I_20_	66:34 M_0_ I_50_	66:34 M_0_ I_80_	66:34 M_1_ I_20_	66:34 M_1_ I_50_	66:34 M_1_ I_80_	
1	*α*-Thujene	921	924	1.02	1.06	0.90	0.80	0.88	0.98	1.05	0.48	1.14	1.26	0.41	1.51	0.98	0.76	1.18	0.44	0.56	1.55	RI-MS
2	*α*-Pinene	931	932	1.63	0.98	1.16	1.12	0.91	0.99	0.92	0.77	1.01	0.82	0.67	0.91	1.04	0.82	1.11	0.94	0.78	1.08	Std
3	Camphene	944	946	1.47	1.73	1.52	1.79	1.43	1.82	1.48	1.14	1.07	1.45	1.28	1.21	1.38	1.06	1.36	1.52	1.14	1.26	Std
4	Sabinene	968	969	0.46	0.29	0.51	0.69	0.39	0.51	0.61	0.37	0.32	0.72	0.31	0.26	0.83	0.39	0.41	0.18	0.22	0.19	Std
5	1-Octen-3-ol	972	974	0.71	0.55	0.58	0.32	0.21	0.22	0.42	0.38	0.52	0.30	0.17	0.14	0.62	0.53	0.53	0.34	0.13	0.17	RI-MS
6	β-Myrcene	985	988	1.69	1.75	1.68	1.56	1.43	1.62	1.84	1.37	1.59	1.46	1.26	1.57	1.55	1.74	1.62	1.44	1.61	1.48	Std
7	3-Octanol	997	998	tr	0.05	–	tr	–	0.05	–	–	–	–	–	tr	tr	tr	–	0.04	–	tr	RI-MS
8	*α*-Terpinene	1011	1014	0.93	0.68	0.83	0.81	0.51	0.67	0.79	0.42	0.73	0.45	0.18	0.43	0.64	0.88	0.70	0.46	0.77	0.59	RI-MS
9	***p*-Cymene**	**1019**	**1020**	**13.07**	**14.73**	**14.04**	**14.48**	**13.76**	**14.79**	**14.86**	**14.12**	**15.44**	**14.09**	**13.85**	**16.06**	**14.89**	**13.88**	**15.83**	**15.34**	**14.33**	**16.26**	Std
10	Limonene	1022	1024	0.67	0.42	0.54	0.61	0.51	0.79	0.50	0.23	0.61	0.59	0.19	0.72	0.88	0.53	0.43	0.77	0.71	0.52	RI-MS
11	1,8-Cineole	1024	1026	1.16	1.41	1.37	0.97	1.12	1.17	1.19	0.99	1.15	0.83	0.94	0.96	1.27	1.25	0.94	0.90	1.06	0.89	Std
12	***γ*-Terpinene**	**1052**	**1054**	**16.93**	**17.96**	**18.12**	**18.42**	**19.06**	**19.12**	**17.02**	**18.46**	**19.14**	**18.54**	**20.88**	**21.55**	**17.13**	**18.73**	**19.44**	**19.53**	**21.37**	**20.63**	Std
13	*cis*-Sabinene hydrate	1064	1065	0.97	0.94	0.92	1.39	0.86	0.86	1.18	0.69	0.83	0.91	0.68	0.77	0.88	0.88	0.91	0.86	0.85	0.82	RI-MS
14	*α*-Terpinolene	1066	1068	0.19	0.12	0.15	0.12	0.08	0.07	0.41	0.09	0.11	0.09	0.09	0.03	0.14	0.16	0.06	0.11	0.08	0.04	RI-MS
15	Linalool	1094	1095	1.82	1.59	1.82	2.16	1.86	2.02	1.81	1.68	1.64	1.95	1.88	1.85	1.87	1.46	1.56	2.06	1.93	1.79	Std
16	Camphor	1137	1141	0.42	0.20	0.26	0.22	0.11	0.22	0.19	0.19	0.20	0.19	0.09	0.07	0.18	0.19	0.20	0.18	0.06	0.08	RI-MS
17	Borneol	1161	1165	1.94	2.12	1.67	1.85	1.37	1.04	2.11	1.67	1.64	1.49	1.15	1.23	2.09	1.49	1.58	1.61	1.02	1.09	Std
18	Terpinen-4-ol	1171	1174	0.53	0.39	0.26	0.52	0.19	0.16	0.93	0.34	0.31	0.46	0.07	0.21	0.73	0.34	0.21	0.29	0.23	0.09	RI-MS
19	Carvacrol, methyl ether	1238	1241	0.82	0.92	0.77	0.59	0.38	0.43	0.78	0.96	0.89	0.48	0.53	0.31	0.78	0.95	0.82	0.37	0.41	0.62	RI–MS
20	Geranial	1260	1264	0.29	0.18	0.13	0.12	0.07	0.06	0.09	tr	0.08	0.09	–	tr	0.09	0.05	0.07	0.09	0.05	–	RI-MS
21	**Thymol**	**1287**	**1289**	**40.89**	**42.23**	**42.46**	**41.17**	**47.29**	**44.09**	**41.32**	**46.05**	**44.12**	**42.71**	**48.59**	**43.13**	**42.28**	**42.66**	**42.56**	**43.87**	**44.58**	**43.16**	Std
22	Carvacrol	1297	1298	2.95	3.62	3.51	3.03	3.16	3.88	3.42	3.58	3.68	3.33	3.52	3.77	3.01	3.91	3.33	3.13	3.46	4.06	Std
23	Thymol acetate	1347	1349	0.08	0.06	0.06	0.11	0.12	0.14	0.12	0.11	0.38	0.12	0.20	0.13	0.02	0.13	0.07	0.09	0.13	0.15	RI-MS
24	(E)-Caryophyllene	1415	1417	1.72	1.27	0.93	2.19	1.65	0.92	1.49	0.59	0.99	2.13	1.82	1.37	1.58	1.26	1.06	2.26	1.11	1.62	Std
25	*α*-Humulene	1451	1452	0.09	0.05	0.04	–	–	–	0.05	tr	–	0.11	tr	–	–	tr	–	–	–	–	RI-MS
26	Germacrene D	1481	1484	0.72	0.86	0.41	0.27	0.12	0.45	0.87	0.29	0.23	0.59	0.06	0.23	0.94	0.41	0.32	0.15	0.11	0.12	RI-MS
27	δ-Cadinene	1521	1522	1.19	0.63	0.92	0.33	0.16	0.14	0.42	0.26	0.18	0.34	0.12	0.18	0.42	0.37	0.24	0.42	0.06	0.24	RI-MS
28	Caryophyllene oxide	1581	1582	1.68	0.94	1.48	0.69	0.62	0.76	1.26	0.76	1.06	0.71	0.59	0.36	1.04	1.15	1.23	0.88	0.81	0.73	Std
29	β-Bisabolene	1766	1768	0.08	0.04	tr	–	–	0.05	0.05	–	tr	–	–	–	tr	0.04	–	–	–	–	RI-MS
Total identified (%)				97.14	99.08	98.06	97.10	98.25	99.02	97.18	98.16	99.09	97.21	98.56	99.13	97.32	98.01	99.77	98.27	99.57	99.25	
**Grouped compounds (%)**																						
Monoterpene hydrocarbons				37.87	39.91	39.3	40.28	41.86	41.29	38.07	37.36	41.05	39.38	39.03	44.06	38.32	40.79	42.08	40.62	43.49	43.56	
Oxygenated monoterpenes				53.06	54.78	54.38	53.25	53.61	55.14	54.55	56.39	55.03	53.65	56.74	52.76	54.34	53.48	52.31	53.56	53.86	52.79	
Sesquiterpene hydrocarbons				3.80	2.85	2.32	2.54	1.95	1.56	2.88	1.17	1.43	3.17	2.03	1.78	2.96	2.04	1.62	2.83	1.28	1.98	
Oxygenated sesquiterpenes				1.68	0.94	1.48	0.69	0.62	0.76	1.26	0.76	1.06	0.71	0.59	0.36	1.04	1.15	1.23	0.88	0.81	0.73	
Others				0.73	0.60	0.58	0.34	0.21	0.27	0.42	0.38	0.52	0.3	0.17	0.17	0.66	0.55	0.53	0.38	0.13	0.19	

^a^RI, linear retention indices on DB-5 MS column, experimentally determined using homologue series of *n*-alkanes0

^b^Relative retention indices taken from Adams.

^c^Ts, 50:50 and 66:34 indicating thyme sole culture and intercropping ratio of thyme with soybean; I_20_, I_50_ and I_80_ indicating non-stressed, moderate and severe water deficit levels; M_0_: non-usage of AMF, M_1_: usage of AMF.

^d^Identification methods: MS, by comparison of the mass spectrum with those of the computer mass libraries Wiley, Adams and NIST 08; RI, by comparison of retention index with those reported in Adams and NIST 08; Std, by comparison of the retention time and mass spectrum of available authentic standard. Bold values show the major constituents of thyme essential oil.

In the first year, the main essential oil constituents were thymol (31.12–37.26%), *γ*-terpinene (13.40–17.48%), *p*-cymene (11.74–13.92%), camphene (6.52–8.51%), 1,8-cineole (2.94–5.83%), camphor (3.74–5.61%), (*E*)-caryophyllene (2.57–4.19%), carvacrol (2.01–3.41%) and *β*-myrcene (1.72–2.31%). In the second year, the main constituents of thyme essential oil were thymol (40.89–48.59%), *γ*-terpinene (16.93–21.55%), *p*-cymene (13.07–16.26%) and carvacrol (2.95–4.06%). The highest content of thymol and *γ*-terpinene was measured in 50:50 intercrop ratio with moderate and severe water deficit under application of AMF. However, the highest content of *p*-cymene was observed in 66:34 intercropping with severe water deficit (I_80_) and application of AMF. In addition, the lowest content of the three mentioned constituents was achieved in the sole culture of thyme and non-stressed plants without AMF. Also, classification of essential oil constituents based on the chemical classes showed that oxygenated monoterpenes and monoterpene hydrocarbons represented the major fraction in the essential oils of thyme. In both years, the highest content of the two mentioned groups was obtained in 50:50 intercrop treated with AMF under moderate and severe water stress (Tables 6 and 7).

## Discussion

The results demonstrated that the root colonization rate increased in intercropping patterns in comparison with sole culture due to an improvement of the soil microbial communities through an enhancement of the organic matter and accessibility of legume-fixed nitrogen into the soil which have a positive effect on root colonization^29^. De Araujo Pereira et al.^30^ showed that the presence of legume species in intercropping patterns could stimulate the alkaline phosphatase activity and increase the AMF root colonization. However, the AMF root colonization of thyme decreased under higher water deficit levels which could be due to the reduction of soil microbial activities under drought conditions. Alike, Shukla et al.^31^ concluded that the different soil conditions, especially moisture, changed the AMF root colonization. These authors noted that the maximum and minimum AMF colonization in the vigna mungo (*Vigna mungo* (L.) Hepper) and wheat (*Triticum aestivum* L.) was observed in the field capacity (FC) and half-field capacity (FC/2), respectively.

Our results showed that the macro- and micro-nutrients of thyme increased in intercropping patterns. The difference in rooting depth and root expansion of plants affects the competition of intercropping components over nutrients. Therefore, the higher nutrient content in intercropping systems could be due to the increase of the environmental use efficiency as a result of different root distribution (e.g., thyme is a shallow-rooted and soybean is a deep-rooted type crop)^32,33^. Duchene et al.^18^ showed that the nutrients availability in intercropping patterns improved due to the higher symbiotic nitrogen fixation by legumes, root exudation of enzymes such as phosphatases, carboxylates, and decrease of soil acidity by production of H^+^ in comparison with sole culture of plants.

Also, the nutrient concentrations of N, P, K, Ca, Mg and Fe improved after application of AMF. The extensive network of AMF hyphae in the plant roots increases the absorption rate and area leading to the enhancement of the micro- and macro-elements uptake, especially lowly mobile nutrients such as P^11^. Alike, Rezaei-Chiyaneh et al.^34^ reported that the nutrients uptake, especially N and P, in intercropping of Isabgol (*Plantago ovata*)/lentil (*Lens culinaris*) significantly increased with AMF inoculation. Also, Weisany et al.^14^ showed that application of AMF (*Funneliformis mosseae*) significantly enhanced the concentration of P, K, Fe, Na, Zn and Mn in the dill/common bean intercrop.

Furthermore, the results of this study exhibited that nutrient concentration decreased by raising the water deficit levels. This could be explained by the reduction of nutrient supply through mineralization, decreasing mass flow and nutrient diffusion that affect the kinetics of nutrient uptake per unit of roots^35^. In addition, Bista et al.^36^ reported that the activity or concentration of major uptake-proteins for nutrients decreased under water stress and had a negative impact on the nutrient’s uptake from roots. Also, these authors noted that the nutrients concentration is higher in the upper soil layers and drought stress reduced the nutrients absorption by decreasing the water uptake in the upper soil layers.

The results showed that dry matter yield of thyme significantly increased under sole culture. In sole culture, the entire of each plot was assigned with one of the plant species. Therefore, the decrease of plants yield in intercrops could be explained by a lower number of partial plants density in these cropping patterns in comparison with sole culture. It seems that the interspecific competition in the thyme/soybean intercrops was higher than the intraspecific competition in sole culture^37^. In addition, the shading of the companion plant (soybean) over thyme seedlings in intercropping patterns could decrease the photosynthetic rate, leading to a decrease in thyme productivity^32^.

The thyme dry matter yield significantly decreased with the increase of water deficit levels. Similar to other plants, thyme plants that tolerate water or drought stresses have apparently evolved mechanisms such as early flowering and structural traits including the reduction of plant leaf area and height and also metabolic responses, such as photosynthetic alterations and proline accumulations (data not shown), to survive under water stress conditions^22^. Therefore, the observed decrease in the thyme growth parameters could be explained by the lower availability of sufficient moisture around the root zone and lower absorption of nutrients as a result of the lower proliferation of root biomass. These conditions had a negative impact on the chlorophyll pigments, the photosynthetic efficiency and finally plant yield^23^. Alike, Askary et al.^4^ showed that total dry matter yield in two different species of thyme (*T. daenensis* Celak. and *T. vulgaris*) decreased by 21.6 and 38.9% in the 67 and 33% FC (field capacity) in comparison with 100% FC, respectively.

Also, our results demonstrated that application of AMF significantly increased the biomass yield of thyme. The mycorrhizal rhizosphere pH is lower due to the absorption of ammonium ions and releasing of H^+^ ions. Reducing soil acidity and also exudation of organic acids and phosphatase enzymes increase the solubility and accessibility of nutrients to plant roots^11^. Therefore, an improvement of plant growth parameters and productivity of thyme after AMF application could be due to the enhancement of the macro- and micronutrients uptake such as N, P, K, Ca, Fe and Mg through the expansion of the mycelium and the development of the root system. Moreover, a higher production of plant growth hormones such as gibberellin (affecting the longitudinal cellular growth and primarily stem internodes), auxin and cytokinin (affecting the cell division) has been observed with AMF inoculation which has positive role in improving plant performance^11,38^. Golubkina et al.^39^ showed that the AMF inoculation increased the biomass and dry yield of *Artemisia dracunculus* L., *Hyssopus officinalis* L. and *Lavandula angustifolia* Mill. compared with untreated plants.

Essential oils are mixtures of low molecular weight compounds that are responsible for the characteristic aroma of medicinal and aromatic plants. The ratio of one special compound or group of compounds to another affects the quality of essential oil in these herbs^3^. Although secondary metabolites such as those contained in medicinal and aromatic plant essential oils are conventionally controlled by the plant genotypes, their biosynthesis is strongly affected by the environmental factors^9^. The obtained results showed that the essential oil content and main constituents (namely thymol, *γ*-terpinene and *p*-cymene) increased in moderate and severe water deficit conditions when compared with non-stressed plants. The increase of essential oil productivity under moderate and severe water stress could be explained by a decrease in the leaf area of plants leading to a raise of the glandular trichomes density and essential oil accumulation per unit of leaf tissue. Also, CO_2_ fixation rate in water stress condition reduced due to decreasing uptake of CO_2_ by closing stomata. In these condition, the ratio of NADPH + H^+^/NADP^+^ enhanced as a result of generating massive oversupply of NADPH + H^+^. It can be concluded that biosynthesis of secondary metabolites such as essential oil, alkaloids, phenols and etc. using additional NADPH + H^+^ known as one of the plant defense mechanism for improving performance in medicinal and aromatic plants^10^. Turtola et al.^40^ noted that the increase of essential oil productivity in medicinal and aromatic plants under drought stress conditions may be related to a low allocation of carbon to the growth, representing a trade-off between growth and plants defense. Govahi et al.^23^ concluded that the maximum essential oil content of sage (*Salvia officinalis* L.) was achieved in moderate drought stress. Also, these authors noted that the essential oil productivity in the moderate and severe drought stress increased by 109 and 84%, compared with non-stressed plants, respectively.

The results showed that the essential oil content and main constituents increased in intercropping patterns with AMF Application. The nutrients accessibility, especially N, plays an important role in the essential oil gland cells size and essential oil productivity in medicinal and aromatic plants^41^. Rostaei et al.^42^ noted that the accessibility of nutrients, especially N and P, play a key role in the development and division of the glandular trichomes, essential oil channels and secretory ducts. In addition, the nutrients availability improves the plant performance and photosynthetic rate in plants. Calsamiglia et al.^43^ noted that availability of glucose in plant cells, which is produced in the process of photosynthesis, enhanced terpenes constituents (especially monoterpenes like thymol) and essential oil productivity in medicinal and aromatic plants. Therefore, the increment of essential oil content and constituents in intercropping patterns with AMF application may be related to the higher accessibility of nutrients by expansion of mycorrhiza hyphae as well as atmospheric nitrogen fixation by legumes and directly/indirectly transfer to component plant (thyme) due to the superior complementarity (special, chemical, and temporal) in intercropping patterns compared with pure cultures^18^. Likewise, Amani Machiani et al.^44^ concluded that the essential oil content and constituents of *Foeniculum vulgare* Mill. and *Dracocephalum moldavica* L. improved in different intercropping patterns with *Phaseolus vulgaris* L. after organic fertilizer usage (humic acid). Rezaei-chiyaneh et al.^45^ showed that different intercropping ratios of fennel/common bean enhance the essential oil content by 24% in comparison with fennel sole culture due to availability of nutrients by nitrogen fixation of legume species.

Interestingly, the thymol content increased by 29.3% in the second year, representing that the thyme essential oil quality was significantly improved in the second year compared with the first year of growing. The quantity and quality of essential oil in medicinal and aromatic plants depends on many factors such as the plant species, climatic conditions, harvest year and plant age. In perennial medicinal plants such as thyme, the first year of planting is considered as the establishment year. In the second year, which is usually considered as the year of economic yield, the plant performance will increase by higher nutrient uptake and photosynthetic rate. Therefore, the improvement of essential oil quantity and quality in aromatic plants during the second year could be explained by the increase of nutrients availability that have positive rule in increasing the oil glands and terpene biosynthesis^46^. Alike, Mechergui et al.^47^ reported that the content of thymol in oregano (*Origanum vulgare* L.) increased in the second year from 31.8 to 41.7% in Tunisian Nefza population and 18.4–31.5% in Tunisian Krib population.

The essential oil yield of thyme increased in the sole culture and decreased after raising the water deficit stress levels. The essential oil yield, calculated from the herbage yield multiply with essential oil content and had a positive relationship with the two mentioned factors. These differences in thyme essential oil yields could be due to the higher dry productivity in the sole culture and application of AMF and to the decrease of the dry productivity under water deficit conditions. In addition, the higher essential oil yield of thyme after application of AMF may be attributed to the higher dry matter and essential oil productivity. Likewise, Bahreininejad et al.^22^ showed that the essential oil yield in *T. daenensis* decreased under moderate and severe water stress due to a decrease of herbage yield in these conditions.

## Conclusions

Our study showed that the water deficit levels decreased dry matter yield of thyme. In contrast, the essential oil content of thyme increased under moderate and severe water deficit conditions. Also, the macro- and micro-nutrients concentration, essential oil content of thyme and main constituents (thymol, *γ*-terpinene and *p*-cymene) increased in intercrop ratio of 50:50 and 66:34 with AMF inoculation. Based on the obtained results, in the semi-arid and arid regions, in which water deficit has a negative impact on the plant productivity, intercropping of thyme with soybean under application of AMF as bio-fertilizer is recommended as a feasible and eco-friendly alternative strategy to improve the thyme essential oil quality and quantity compared with the sole culture.

## References

[1] Tohidi B, Rahimmalek M, Trindade H (2019). Review on essential oil, extracts composition, molecular and phytochemical properties of Thymus species in Iran. Ind. Crops Prod..

[2] Alavi-Samani SM, Kachouei MA, Pirbalouti AG (2015). Growth, yield, chemical composition, and antioxidant activity of essential oils from two thyme species under foliar application of jasmonic acid and water deficit conditions. Hortic. Environ. Biotechnol..

[3] Lubbe A, Verpoorte R (2011). Cultivation of medicinal and aromatic plants for specialty industrial materials. Ind. Crops Prod..

[4] Askary M, Behdani MA, Parsa S, Mahmoodi S, Jamialahmadi M (2018). Water stress and manure application affect the quantity and quality of essential oil of *Thymus daenensis* and *Thymus vulgaris*. Ind. Crops Prod..

[5] Hosseinzadeh S, Kukhdan A, Hosseini A, Armand R (2015). The application of *Thymus vulgaris* in traditional and modern medicine: A review. Glob. J. Pharmacol..

[6] Pavela R, Žabka M, Vrchotová N, Tříska J (2018). Effect of foliar nutrition on the essential oil yield of Thyme (*Thymus vulgaris* L.). Ind. Crops Prod..

[7] Okunlola GO, Olatunji OA, Akinwale RO, Tariq A, Adelusi AA (2017). Physiological response of the three most cultivated pepper species (*Capsicum* spp.) in Africa to drought stress imposed at three stages of growth and development. Sci. Hortic..

[8] Gao S, Wang Y, Yu S, Huang Y, Liu H, Chen W, He X (2020). Effects of drought stress on growth, physiology and secondary metabolites of Two Adonis species in Northeast China. Sci. Hortic..

[9] Morshedloo MR, Salami SA, Nazeri V, Craker LE (2017). Prolonged water stress on growth and constituency of Iranian of Oregano (*Origanum vulgare* L.). J. Med. Act. Plants..

[10] Kleinwächter M, Selmar D (2015). New insights explain that drought stress enhances the quality of spice and medicinal plants: Potential applications. Agron. Sustain. Dev..

[11] Begum N, Qin C, Ahanger MA, Raza S, Khan MI, Ashraf M, Ahmed N, Zhang L (2019). Role of arbuscular mycorrhizal fungi in plant growth regulation: Implications in abiotic stress tolerance. Front. Plant Sci..

[12] El-Nashar YI, Hassan BA, Aboelsaadat EM (2021). Response of Nemesia (*Nemesia* × *hybridus*) plants to different irrigation water sources and arbuscular mycorrhizal fungi inoculation. Agric. Water Manag..

[13] Ostadi A, Javanmard A, Amani Machiani M, Morshedloo MR, Nouraein M, Rasouli F, Maggi F (2020). Effect of different fertilizer sources and harvesting time on the growth characteristics, nutrient uptakes, essential oil productivity and composition of *Mentha × piperita* L.. Ind. Crops Prod..

[14] Weisany W, Raei Y, Salmasi SZ, Sohrabi Y, Ghassemi-Golezani K (2016). Arbuscular mycorrhizal fungi induced changes in rhizosphere, essential oil and mineral nutrients uptake in dill/common bean intercropping system. Ann. Appl. Biol..

[15] Malézieux E, Crozat Y, Dupraz C, Laurans M, Makowski D, Ozier-Lafontaine H, Rapidel B, Tourdonnet S, Valantin-Morison M (2009). Mixing plant species in cropping systems: Concepts, tools and models. A review. Agron. Sustain. Dev..

[16] Gomiero T, Pimentel D, Paoletti MG (2011). Environmental impact of different agricultural management practices: Conventional vs. organic agriculture. CRC Crit. Rev. Plant Sci..

[17] Yin W, Chai Q, Zhao C, Yu A, Fan Z, Hu F, Fan H, Guo Y, Coulter JA (2020). Water utilization in intercropping: A review. Agric. Water Manag..

[18] Duchene O, Vian JF, Celette F (2017). Intercropping with legume for agroecological cropping systems: Complementarity and facilitation processes and the importance of soil microorganisms. A review. Agric. Ecosyst. Environ..

[19] Amani Machiani M, Javanmard A, Morshedloo MR, Maggi F (2018). Evaluation of competition, essential oil quality and quantity of peppermint intercropped with soybean. Ind. Crops Prod..

[20] Gong X, Dang K, Liu L, Zhao G, Lv S, Tian L, Jin F, Feng Y, Zhao Y, Feng B (2021). Intercropping combined with nitrogen input promotes proso millet (*Panicum miliaceum* L.) growth and resource use efficiency to increase grain yield on the Loess plateau of China. Agric. Water Manag..

[21] Biglari T, Maleksaeidi H, Eskandari F, Jalali M (2019). Livestock insurance as a mechanism for household resilience of livestock herders to climate change: Evidence from Iran. Land Use Policy.

[22] Bahreininejad B, Razmjoo J, Mirza M (2013). Influence of water stress on morpho-physiological and phytochemical traits in *Thymus daenensis*. Int. J. Plant Prod..

[23] Govahi M, Ghalavand A, Nadjafi F, Sorooshzadeh A (2015). Comparing different soil fertility systems in Sage (*Salvia officinalis*) under water deficiency. Ind. Crops Prod..

[24] Phillips JM, Hayman D (1970). Improved procedures for clearing roots and staining parasitic and vesicular-arbuscular mycorrhizal fungi for rapid assessment of infection. Trans. Br. Mycol. Soc..

[25] Giovannetti M, Mosse B (1980). An evaluation of techniques for measuring vesicular arbuscular mycorrhizal infection in roots. New Phytol..

[26] Adams, R. P. *Identification of Essential Oil Components by Gascromatography/Quadrupole Mass Spectroscopy*, 4th ed. 455 (Allured Publishing Corporation, 2007).

[27] Jones JB, Mortvedt JJ (1972). Plant tissue analysis for micronutrients. Micronutrients in Agriculture.

[28] Tandon HLS, Cescas MP, Tyner EH (1968). An acid-free vanadate-molybdate reagent for the determination of total phosphorus in soils1. Soil Sci. Soc. Am. J..

[29] Wahbi S, Maghraoui T, Hafidi M, Sanguin H, Oufdou K, Prin Y, Duponnois R, Galiana A (2016). Enhanced transfer of biologically fixed N from faba bean to intercropped wheat through mycorrhizal symbiosis. Appl. Soil Ecol..

[30] De Araujo Pereira AP, Santana MC, Bonfim JA, de Lourdes Mescolotti D, Cardoso EJBN (2018). Digging deeper to study the distribution of mycorrhizal arbuscular fungi along the soil profile in pure and mixed *Eucalyptus grandis* and *Acacia mangium* plantations. Appl. Soil Ecol..

[31] Shukla A, Kumar A, Jha A, Salunkhe O, Vyas D (2013). Soil moisture levels affect mycorrhization during early stages of development of agroforestry plants. Biol. Fertil. Soils..

[32] Amani Machiani M, Javanmard A, Morshedloo MR, Maggi F (2018). Evaluation of yield, essential oil content and compositions of peppermint (*Mentha piperita* L.) intercropped with faba bean (*Vicia faba* L.). J. Clean. Prod..

[33] Maitra S, Hossain A, Brestic M, Skalicky M, Ondrisik P, Gitari H, Brahmachari K, Shankar T, Bhadra P, Palai JB, Jena J, Bhattacharya U, Duvvada SK, Lalichetti S, Sairam M (2021). Intercropping—A low input agricultural strategy for food and environmental security. Agronomy.

[34] Rezaei-Chiyaneh E, Jalilian J, Seyyedi SM, Barin M, Ebrahimian E, Afshar RK (2021). Isabgol (*Plantago ovata*) and lentil (*Lens culinaris*) intercrop responses to arbuscular mycorrhizal fungi inoculation. Biol. Agric. Hortic..

[35] Hussain HA, Hussain S, Khaliq A, Ashraf U, Anjum SA, Men S, Wang L (2018). Chilling and drought stresses in crop plants: Implications, cross talk, and potential management opportunities. Front. Plant Sci..

[36] Bista DR, Heckathorn SA, Jayawardena DM, Mishra S, Boldt JK (2018). Effects of drought on nutrient uptake and the levels of nutrient-uptake proteins in roots of drought-sensitive and -tolerant grasses. Plants..

[37] Xie Y, Kristensen HL (2017). Intercropping leek (*Allium porrum* L.) with dyer’s woad (*Isatis tinctoria* L.) increases rooted zone and agro-ecosystem retention of nitrogen. Eur. J. Agron..

[38] Hashem A, Alqarawi AA, Radhakrishnan R, Al-Arjani ABF, Aldehaish HA, Egamberdieva D, Abd Allah EF (2018). Arbuscular mycorrhizal fungi regulate the oxidative system, hormones and ionic equilibrium to trigger salt stress tolerance in *Cucumis sativus* L.. Saudi J. Biol. Sci..

[39] Golubkina N, Logvinenko L, Novitsky M, Zamana S, Sokolov S, Molchanova A, Shevchuk O, Sekara A, Tallarita A, Caruso G (2020). Yield, essential oil and quality performances of *artemisia dracunculus*, *hyssopus officinalis* and *lavandula angustifolia* as affected by arbuscular mycorrhizal fungi under organic management. Plants..

[40] Turtola S, Manninen AM, Rikala R, Kainulainen P (2003). Drought stress alters the concentration of wood terpenoids in Scots pine and Norway spruce seedlings. J. Chem. Ecol..

[41] Rehman R, Hanif MA, Mushtaq Z, Al-Sadi AM (2016). Biosynthesis of essential oils in aromatic plants: A review. Food Rev..

[42] Rostaei M, Fallah S, Lorigooini Z, Abbasi Surki A (2018). The effect of organic manure and chemical fertilizer on essential oil, chemical compositions and antioxidant activity of dill (*Anethum graveolens*) in sole and intercropped with soybean (*Glycine max*). J. Clean. Prod..

[43] Calsamiglia S, Busquet M, Cardozo PW, Castillejos L, Ferret A (2007). Invited review: Essential oils as modifiers of rumen microbial fermentation. J. Dairy Sci..

[44] Amani Machiani M, Rezaei-Chiyaneh E, Javanmard A, Maggi F, Morshedloo MR (2019). Evaluation of common bean (*Phaseolus vulgaris* L.) seed yield and quali-quantitative production of the essential oils from fennel (*Foeniculum vulgare* Mill.) and dragonhead (*Dracocephalum moldavica* L.) in intercropping system under humic acid application. J. Clean. Prod..

[45] Rezaei-Chiyaneh E, Amirnia R, Amani Machiani M, Javanmard A, Maggi F, Morshedloo MR (2020). Intercropping fennel (*Foeniculum vulgare* L.) with common bean (*Phaseolus vulgaris* L.) as affected by PGPR inoculation: A strategy for improving yield, essential oil and fatty acid composition. Sci. Hortic..

[46] Emami Bistgani Z, Ataollah Siadat S, Bakhshandeh A, Ghasemi Pirbalouti A, Hashemi M, Maggi F, Morshedloo MR (2018). Application of combined fertilizers improves biomass, essential oil yield, aroma profile, and antioxidant properties of *Thymus daenensis* Celak. Ind. Crops Prod..

[47] Mechergui K, Jaouadi W, Coelho JP, Khouja ML (2016). Effect of harvest year on production, chemical composition and antioxidant activities of essential oil of oregano (*Origanum vulgare* subsp glandulosum (Desf.) Ietswaart) growing in North Africa. Ind. Crops Prod..

